# SUVmax—Δ makes the difference

**DOI:** 10.1093/icvts/ivac169

**Published:** 2022-06-24

**Authors:** Clemens Aigner, Hubertus Hautzel, Till Ploenes

**Affiliations:** Department of Thoracic Surgery, West German Cancer Center, University Medicine Essen, Essen, Germany; Department of Nuclear Medicine, West German Cancer Center, University Medicine Essen, Essen, Germany; Department of Thoracic Surgery, West German Cancer Center, University Medicine Essen, Essen, Germany

**Keywords:** SUVmax, Non-small cell lung cancer, Metabolic response, Neoadjuvant, Chemoradiotherapy, Surgery, Stage III

The authors must be congratulated on systematically assessing the role of fluorodeoxyglucose-positron emission tomography and computed tomography in predicting pathological response and prognosis of locally advanced non-small cell lung cancer (NSCLC) after neoadjuvant chemoradiotherapy [1]. Integrated ^18^F-fluorodeoxyglucose-positron emission tomography is the standard in pre-therapeutic staging of NSCLC recommended by recent guidelines. A decrease in SUV max of more than 60% in the primary tumour was identified as optimal cut-off value for predicting major pathological response and performed better than computed tomography morphologic partial response measured by a size reduction of the primary tumour of more than 30%. The same result was observed for prediction of prognosis.

Relevant differences were identified for the subgroup of T3-4N0-1 patients compared to N2 positive patients and a decrease of <60% of SUVmax was found as an indicator of poor prognosis, particularly in N2 patients. The analysis was focused on metabolic and imaging response in the primary tumour, whereas lymph node status was analysed pathologically.

Previous analyses of well-designed prospective trials looking at pre-treatment metabolic tumour volume only showed conflicting results on the impact on 5-year survival rates [2], whereas metabolic response as derived from dual positron emission tomography/computed tomography pre- and post-induction chemotherapy has been demonstrated to be a prognostic classifier [3]. Regarding the effects of neoadjuvant chemotherapy only further studies indicate that Δ SUVmax and SUV-derived measures like Δ metabolic tumour volume and Δ total lesion glycolysis are suitable tools for response and outcome prediction in NSCLC patients [4, 5]. However, additional data on the performance of metabolic response rates for predicting pathologic response rates and long-term outcome after neoadjuvant chemoradiotherapy in lung cancer patients are valuable, particularly to provide reference data of established chemoradiotherapy schemes in view of the constantly evolving neoadjuvant treatment options. Due to the retrospective design of the study no standardized timepoint of performing the positron emission tomography scan after induction treatment was used; however, the timeframe of 2–4 weeks is reasonable to ensure sufficient homogeneity in view of available previous data.

In the current treatment landscape, the presented data will be challenged by several issues. Particularly, the details of pathologic reporting after neoadjuvant treatment are crucial to ensure comparability of trials. In 2020, the ‘International Association for the Study of Lung Cancer’ (IASLC) has reported a multidisciplinary recommendation for pathologic assessment of lung cancer resection specimens after neoadjuvant treatment [6]. The authors use the Japanese Lung Cancer Society recommendation [7], which varies considerably from the IASLC recommendations in the definition of major and minor pathological response. Thus, care must be taken when comparing results to other recently published trials using a different definition.

The integration of immunotherapy in neoadjuvant treatment algorithms [8] will lead to a substantial shift in interpretation of positron emission tomography/computed tomography imaging for restaging due to phenomenons like pseudoprogression and nodal immune flare [9, 10]. Additionally, the integration of targeted therapies in curative stages considerably impacts the prognosis of patients in an adjuvant setting and is investigated in neoadjuvant trials as well.

In summary, innovative neoadjuvant combination schemes have the potential to substantially change staging algorithms in NSCLC. The paper by Tanahashi and colleagues provides relevant confirmatory reference data on the impact of metabolic response on pathologic response rates and 5-year survival rates after neoadjuvant chemoradiation therapy.
